# Clinico-Pathological Discordance in a Partial Hydatidiform Mole With Coexisting Live Fetus and Subsequent Aggressive Gestational Trophoblastic Neoplasia: A Case Report

**DOI:** 10.7759/cureus.111624

**Published:** 2026-06-27

**Authors:** Giancarlo A. Turri-Vasquez, Gabriel A Eman-Greci, Steffany Castro-El Fakhri, Daniel Patino-Sepulveda, Vicente Bosque, Rafael Cortes-Charry

**Affiliations:** 1 Department of Obstetrics and Gynecology, Hospital Universitario de Caracas, Caracas, VEN; 2 Department of Minimally Invasive Gynecologic Surgery and Pelvic Floor Surgery, Centro Médico Docente La Trinidad, Caracas, VEN

**Keywords:** drug resistance neoplasm, gestational trophoblastic neoplasia, ovarian torsion, partial hydatidiform mole, pregnancy twin

## Abstract

Twin pregnancies with a partial hydatidiform mole and a coexisting live fetus are exceptionally rare, and progression to chemoresistant gestational trophoblastic neoplasia (GTN) is even less common.

We report the case of a 28-year-old nulligravid woman with prior ovulation induction who presented with a twin pregnancy consisting of a partial hydatidiform mole and a coexisting live normal fetus. Following spontaneous abortion and uterine evacuation, histopathological examination and immunohistochemistry demonstrated p57 positivity, supporting the diagnosis of partial hydatidiform mole, and a normal placental tissue with a fetus without malformations. Despite apparently favorable pathological findings, the patient developed giant theca-lutein cysts complicated by ovarian torsion, progression to GTN, resistance to methotrexate and actinomycin-D, and subsequent pulmonary metastasis. Multi-agent chemotherapy with EMA-CO (etoposide, methotrexate, actinomycin-D, cyclophosphamide, and vincristine) achieved complete clinical and biochemical remission, with no evidence of recurrence after three years of follow-up. This case highlights that partial hydatidiform mole with a coexisting live fetus may rarely exhibit aggressive clinical behavior despite reassuring histopathological and immunohistochemical features. Careful clinical and human chorionic gonadotropin surveillance remains essential in complex molar pregnancies.

## Introduction

Gestational trophoblastic neoplasia (GTN) comprises a spectrum of premalignant and malignant placental disorders, including complete and partial hydatidiform mole (PHM) [1,2]. PHM is typically associated with a malformed fetus or embryonic tissue and generally carries a lower risk of progression to GTN than complete hydatidiform mole [1]. Twin pregnancies with a molar component and a coexisting live fetus are rare, with an estimated incidence ranging from 1 in 22,000 to 1 in 100,000 pregnancies; most reported cases involve complete hydatidiform mole, whereas PHM with a coexisting live fetus is exceptionally uncommon [3].

We report a twin pregnancy with PHM and a coexisting pregnancy with a live fetus that progressed to chemoresistant metastatic GTN despite apparently favorable histopathological and immunohistochemical findings. This case highlights the potential discordance between pathological features and clinical behavior in complex molar pregnancies and reinforces the importance of close clinical and human chorionic gonadotropin (hCG) surveillance regardless of initial pathological assessment.

## Case presentation

A 28-year-old nulligravid woman from Caracas, Venezuela, with no significant medical or surgical history, presented with infertility treated with ovulation induction using follitropin alfa. She subsequently achieved her first pregnancy.

At 12 weeks and 5 days of gestation, according to the last menstrual period, she presented to the emergency department with mild vaginal bleeding. She was hemodynamically stable, and an obstetric ultrasound initially demonstrated a single live fetus without malformations and preserved fetal cardiac activity. She was discharged with outpatient management for a threatened abortion.

**Figure 1:**
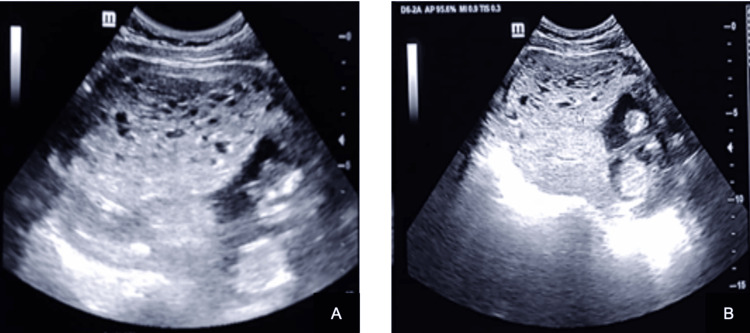
Obstetric ultrasound findings Ultrasound images demonstrating a multicystic placental mass with a “snowstorm” appearance (A) coexisting with a live fetus (B), findings suggestive of molar pregnancy.

Five days later, she returned because of increased vaginal bleeding and right lower quadrant abdominal pain. Serum hCG level was 353,800 mIU/mL, while thyroid function tests were within normal limits. The obstetric ultrasound demonstrated gestation with a live fetus coexisting with a multicystic placental mass with a “snowstorm” appearance, separate from an apparently normal fetal placenta (Figures 1A-1B).

The patient subsequently developed an incomplete spontaneous abortion requiring uterine evacuation by suction curettage and dilation.

**Figure 2:**
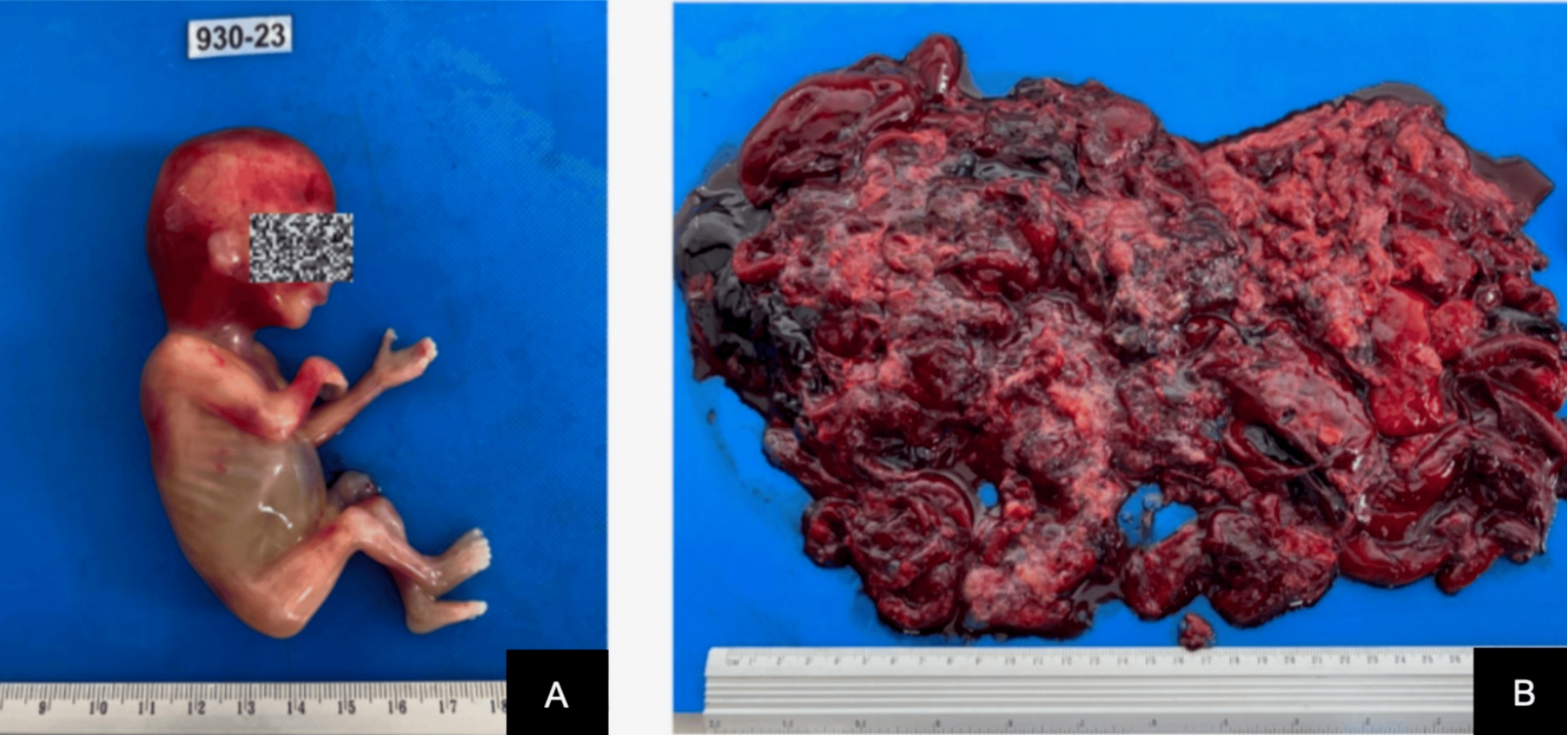
Macroscopic findings after uterine evacuation Gross pathological specimens obtained after uterine evacuation demonstrating the coexisting fetus from the twin gestation (A) and the molar component with placental tissue (B).

Gross pathological examination confirmed twin gestation with two distinct placental components: a male fetus weighing 57 g without apparent external or internal malformations (Figure 2A), and a molar mass weighing 605 g with multiple hydropic vesicles and a separate discoid placenta weighing 17 g (Figure 2B).

**Figure 3:**
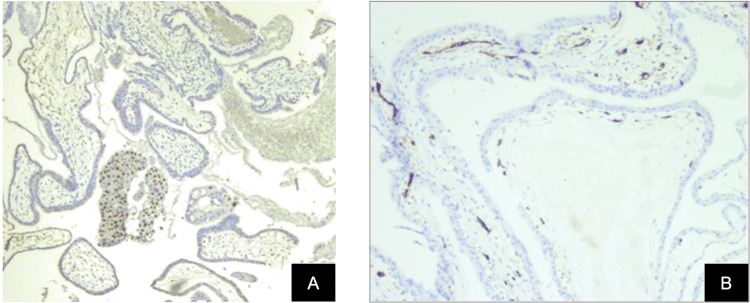
p57 immunohistochemical staining (200×) and CD34 immunohistochemical staining (200×) (A) Immunohistochemical staining demonstrating nuclear p57 positivity in cytotrophoblast cells within chorionic villi. (B) Immunohistochemical staining demonstrating positive CD34 expression in villous vascular endothelium within chorionic villi.

Histopathological examination demonstrated chorionic villi of variable size with hydropic degeneration and cistern formation alternating with smaller fibrotic villi. Immunohistochemistry showed nuclear p57 positivity in cytotrophoblast cells and positive CD34 staining in villous vascular walls (Table 1; Figures 3A-3B).

**Table 1:** Immunohistochemical findings

Marker	Result
p57	Nuclear positivity in cytotrophoblast cells
PLAP	Positive in syncytiotrophoblast
β-hCG	Positive in syncytiotrophoblast
CD34	Positive staining in villous vascular walls
Ki-67	Proliferation index <5%

The Ki-67 proliferation index was below 5%. Fetal and trophoblastic karyotyping could not be performed because of financial limitations.

**Figure 4:**
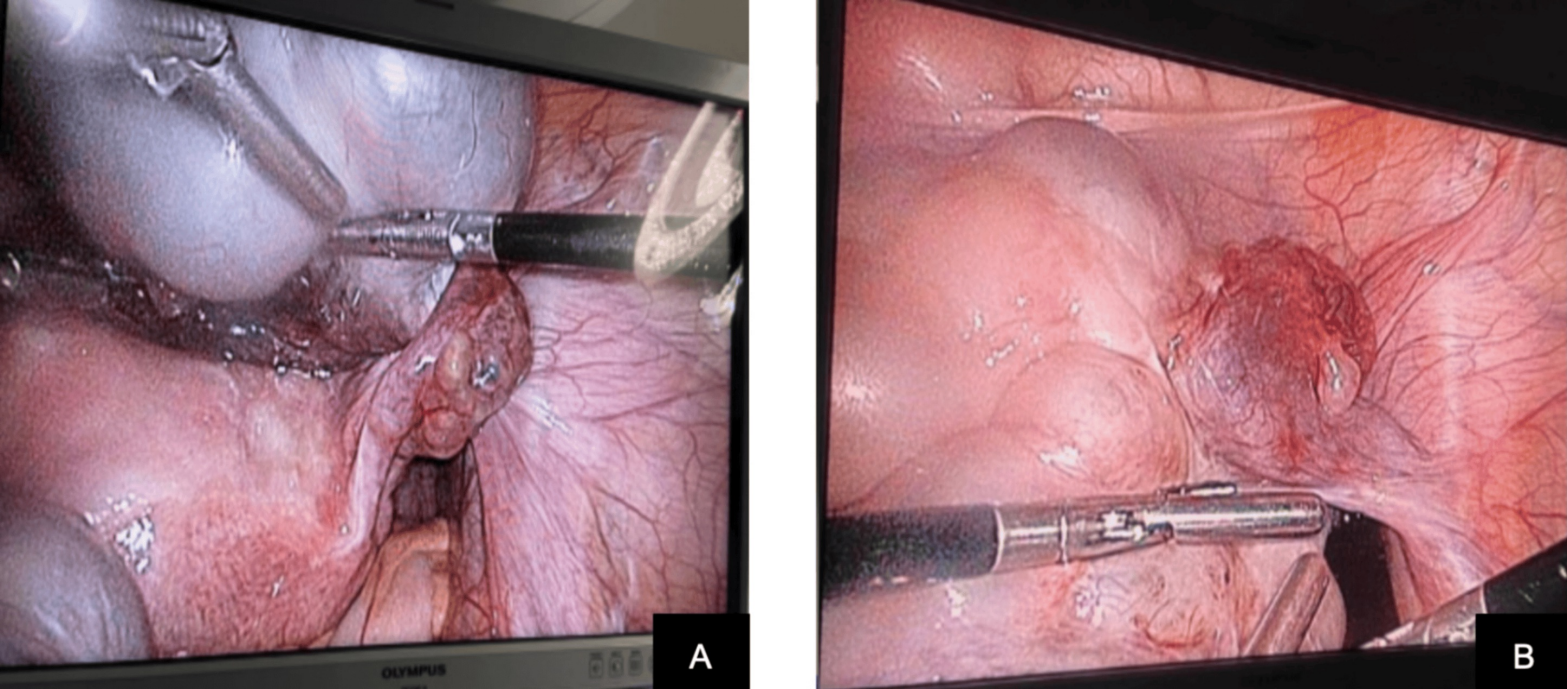
Laparoscopic findings of ovarian torsion (A) Right ovarian torsion secondary to giant theca-lutein cysts. (B) Laparoscopic appearance after conservative detorsion with adnexal preservation.

Three days after uterine evacuation, serum hCG increased to 1,290,000 mIU/mL. Ten days later, the patient developed acute abdominal pain requiring emergency laparoscopy, which demonstrated right ovarian torsion secondary to giant theca-lutein cysts (Figures 4A-4B).

Conservative detorsion and ovarian cystectomy with adnexal preservation were performed.

One month after evacuation, she presented with profuse vaginal bleeding. Serum hCG level was 38,320 mIU/mL, and transvaginal ultrasound demonstrated a 30-mm hypervascular myometrial nodule in the uterine fundus, suggestive of myometrial invasion. Computed tomography imaging initially showed no evidence of metastatic disease, and GTN stage I with FIGO (International Federation of Gynecology and Obstetrics) risk score 2 was diagnosed.

First-line treatment with methotrexate and folinic acid rescue was initiated. Despite treatment, serial hCG measurements demonstrated biochemical resistance. Follow-up chest radiography subsequently revealed two right pulmonary nodules consistent with metastatic progression, leading to restaging as stage III disease. 

Second-line treatment with actinomycin-D was administered; however, hCG levels remained persistently elevated above 1,000 mIU/mL, indicating sequential resistance to single-agent chemotherapy. Multi-agent chemotherapy with EMA-CO (etoposide, methotrexate, actinomycin-D, cyclophosphamide, and vincristine) was therefore initiated. The patient completed six cycles and experienced moderate alopecia, nausea, and photosensitivity managed symptomatically.

Complete clinical and biochemical remission was achieved, with post-treatment hCG levels of 2.3 mIU/mL and negative positron emission tomography-computed findings. Monthly hCG surveillance was performed for one year. Following standard protocols, the patient was prescribed progestin-only oral contraceptives during the entire treatment course and the subsequent one-year surveillance period. She was strictly counseled to defer attempts at conception for at least 12 months after achieving complete remission to prevent unwanted pregnancies, ensure accurate serial hCG monitoring, and reliably rule out disease recurrence. After three years of follow-up, the patient remains asymptomatic with no evidence of recurrent disease.

## Discussion

The most notable feature of this case was the marked discordance between the apparently favorable pathological findings and the subsequent aggressive clinical course. PHM is generally associated with a lower risk of progression to GTN than complete hydatidiform mole [1]. In this patient, immunohistochemistry demonstrated nuclear p57 positivity and a low Ki-67 proliferation index, findings usually considered reassuring. Nevertheless, the patient developed persistent GTN with pulmonary metastasis and sequential resistance to methotrexate and actinomycin-D.

Twin pregnancies with a molar component and a coexisting live fetus are rare, and most reported cases involve complete hydatidiform mole [3]. Cases involving PHM with a coexisting live fetus progressing to metastatic or chemoresistant GTN are exceptionally uncommon [1]. Gajewska et al. described a similar case in which a twin pregnancy with PHM and a live fetus evolved into metastatic GTN despite initially favorable pathological findings [4]. Together, these observations suggest that histopathological and immunohistochemical features alone may not reliably predict biological behavior in complex molar pregnancies. 

This case also highlights the importance of close hCG surveillance and careful clinical follow-up regardless of apparently favorable pathology. The patient presented with markedly elevated hCG levels and giant theca-lutein cysts complicated by ovarian torsion, findings previously associated with increased risk of post-molar GTN [5,6]. Despite initial classification as low-risk GTN according to FIGO criteria [7], she ultimately required multiagent chemotherapy with EMA-CO to achieve complete remission.

An important limitation of this report was the inability to perform fetal and trophoblastic karyotyping because of financial constraints, which prevented confirmation of ploidy and exclusion of confined placental mosaicism. Nevertheless, the clinical, histopathological, and immunohistochemical findings strongly supported the diagnosis of PHM with a coexisting live fetus.

## Conclusions

This case demonstrates that a partial hydatidiform mole with a coexisting live fetus may rarely exhibit aggressive clinical behavior, including metastatic and chemoresistant gestational trophoblastic neoplasia, despite apparently favorable histopathological and immunohistochemical findings. Clinical management and post-evacuation surveillance should therefore be guided by the overall clinical course and serial hCG monitoring rather than pathological features alone.

## References

[1] Ngan HY, Seckl MJ, Berkowitz RS (2021). Diagnosis and management of gestational trophoblastic disease: 2021 update. Int J Gynaecol Obstet.

[2] Hui P (2019). Gestational trophoblastic tumors: a timely review of diagnostic pathology. Arch Pathol Lab Med.

[3] Lin LH, Maestá I, Braga A (2017). Multiple pregnancies with complete mole and coexisting normal fetus in North and South America: a retrospective multicenter cohort and literature review. Gynecol Oncol.

[4] Gajewska M, Zygula A, Wielgos M, Szewczyk G (2020). Twin pregnancy with a partial hydatidiform mole and a coexistent live fetus. Diagnostic and therapeutic dilemmas. A case report and the review of literature. Ginekol Pol.

[5] Massardier J, Golfier F, Journet D (2009). Twin pregnancy with complete hydatidiform mole and coexistent fetus: obstetrical and oncological outcomes in a series of 14 cases. Eur J Obstet Gynecol Reprod Biol.

[6] Piazzetta SR, Hoch KA, Benetti-Pinto CL, Yela DA (2024). Assessment of risk factors associated with post-molar gestational trophoblastic neoplasia: a retrospective cohort. Rev Bras Ginecol Obstet.

[7] Mangili G, Lorusso D, Brown J (2014). Trophoblastic disease review for diagnosis and management: a joint report from the International Society for the Study of Trophoblastic Disease, European Organisation for the Treatment of Trophoblastic Disease, and the Gynecologic Cancer InterGroup. Int J Gynecol Cancer.

